# Hardware Accelerators for Cardiovascular Signal Processing: A System-on-Chip Perspective

**DOI:** 10.3390/mi17010051

**Published:** 2025-12-30

**Authors:** Rami Hariri, Marcian Cirstea, Mahdi Maktab Dar Oghaz, Khaled Benkrid, Oliver Faust

**Affiliations:** 1 School of Computing and Information Science, Anglia Ruskin University, East Rd., Cambridge CB1 1PT, UK; 2 Arm Ltd., Cambridge CB1 9NJ, UK

**Keywords:** cardiovascular disease, electrocardiogram, photoplethysmogram, machine learning, deep learning, field programmable gate array, application specific integrated circuit

## Abstract

This study presents a comprehensive systematic analysis, investigating hardware accelerators specifically designed for real-time cardiovascular signal processing, focusing mainly on Electrocardiogram (ECG), Photoplethysmogram (PPG), and blood pressure monitoring systems. Cardiovascular Diseases (CVDs) represent the world’s leading cause of morbidity and mortality, creating an urgent demand for efficient and accurate diagnostic technologies. Following the Preferred Reporting Items for Systematic Reviews and Meta-Analyses (PRISMA) guidelines, we systematically analysed 59 research papers on this topic, published from 2014 to 2024, categorising them into three main categories: signal denoising, feature extraction, and decision support with Machine Learning (ML) or Deep Learning (DL). A comprehensive performance benchmarking across energy efficiency, processing speed, and clinical accuracy demonstrates that hybrid Field Programmable Gate Array (FPGA)-Application Specific Integrated Circuit (ASIC) architectures and specialised Artificial Intelligence (AI) on Edge accelerators represent the most promising solutions for next-generation CVD monitoring systems. The analysis identifies key technological gaps and proposes future research directions focused on developing ultra-low-power, clinically robust, and highly scalable physiological signal processing systems. The findings provide guidance for advancing hardware-accelerated cardiovascular diagnostics toward practical clinical deployment.

## 1. Introduction

Over 17.9 million people die from Cardiovascular Diseases (CVDs) every year [1], making them one of the leading causes of morbidity and mortality worldwide. According to the American Heart Association [2], CVD-related deaths are projected to increase to 23 million by 2030. The economic burden is also significant: in 2019, 12% of total healthcare expenditures in the United States, amounting to USD 422 billion, were attributed to CVDs [3]. Globally, this figure is expected to rise to USD 1.44 trillion annually by 2050 [4]. Early diagnosis and treatment are essential in reducing this burden. Evidence shows a strong correlation between early detection and improved treatment outcomes [5]. In many cases, early intervention requires only minimally invasive measures or simple lifestyle changes [6].

Continuous real-time monitoring is critical for the early detection and diagnosis of CVDs, driving demand for efficient signal processing solutions [7]. Among the most widely used techniques are Electrocardiogram (ECG), Photoplethysmogram (PPG), and blood pressure monitoring [8]. These signals reflect the heart’s electrical and circulatory activities, enabling the detection of conditions like arrhythmias and myocardial infarctions. However, raw physiological signals require sophisticated processing to extract clinically actionable insights. Software-only processing approaches often struggle with speed, power efficiency, and portability constraints. These limitations become particularly apparent in real-time medical applications. While offering flexibility, software solutions typically require transmitting raw data to external devices or cloud servers, introducing latency and high energy consumption [9]. These inefficiencies become critical barriers for wearable devices and remote monitoring systems where timely intervention depends on low-latency processing [10].

Hardware accelerators like Field Programmable Gate Arrays (FPGAs) and Application Specific Integrated Circuits (ASICs), have emerged as compelling solutions by enabling parallel, on-chip processing. Three key advantages distinguish hardware approaches: superior processing speed through parallel logic architectures [11]; reduced power consumption via optimised resource allocation and slower clock speeds [12]; and enhanced portability through compact designs suitable for wearable applications [5]. For example, hardware implementations can outperform software in both speed and energy efficiency, even when operating at lower clock frequencies, making them ideal for continuous monitoring scenarios.

The rapid diversification of hardware approaches, from FPGA-based ECG analysers to ASIC-optimised PPG pipelines, reflects both innovation and fragmentation in the field. This diversity stems from varying clinical requirements, signal characteristics (e.g., the high data rate of ECG versus lower-bandwidth Heart Rate Variability (HRV) signals [5]), and implementation constraints. Such variation underscores the need for a comprehensive analysis that evaluates trade-offs in processing architectures, benchmarks performance metrics, and identifies optimal solutions for specific CVD monitoring applications.

Analysing hardware accelerators for cardiovascular signal processing is particularly relevant today, as it helps to contextualise these technologies within the wider spectrum of solutions addressing specific physiological and clinical needs. It also helps to understand the role of emerging enabler technologies that support their development and deployment. During the analysis we learned that the evolving landscape of healthcare needs demands efficient, real-time, and accurate diagnostic devices for CVDs. Progress in miniaturised electronics has led to the creation of compact, wearable, and even implantable devices for continuous heart monitoring. In addition, significant progress has been made in Artificial Intelligence (AI) and Machine Learning (ML) algorithms, particularly Deep Learning (DL), for signal analysis and anomaly detection. These algorithms are computationally demanding, but they are essential for advanced health monitoring. Furthermore, the demand for Edge computing, known as AI at Edge, driven by the need to shift data processing closer to the source, has provided essential benefits in terms of real-time performance, enhanced privacy, and improved energy efficiency. Such capabilities are particularly valuable for continuous cardiac health monitoring applications. The convergence of these advancements highlights the growing need to evaluate how hardware acceleration has impacted cardiovascular signal processing. This analytical review systematically examines hardware acceleration techniques for processing key cardiovascular signals, including ECG, PPG, and blood pressure waveforms. It focuses on acceleration methods used for noise reduction, feature extraction, and disease classification, such as arrhythmia detection, blood pressure estimation, and sleep stage analysis. The analysis surveys solutions implemented using FPGAs, ASICs, Graphics Processing Units (GPUs), and specialised embedded Digital Signal Processings (DSPs) or AI accelerators, specifically targeting real-time, low-power applications at the Edge. Our analysis includes 59 peer-reviewed academic publications that develop or implement dedicated hardware solutions for cardiovascular signal processing. These works fall into three primary categories: Decision support with ML or DL (9 publications), Feature extraction (22 publications), and Denoising/Filtering (28 publications). To synthesise these diverse contributions into a coherent framework, we introduce a dedicated taxonomy that links target function, hardware technology, and algorithmic approach. This taxonomy structures the organisation of the subsequent sections and enables a consistent comparison across the reviewed accelerator designs. Based on the structured results we highlight key trends, technological trade-offs, and research gaps in the field of hardware acceleration for cardiovascular applications.

This paper is structured as follows: Section 2 provides background on cardiovascular signals and processing needs. Section 3 presents the methodology adopted to perform the critical analysis. Section 4 discusses the results, while Section 5 explores current limitations and future research directions. Finally, Section 6 concludes with a summary of key findings and highlights open challenges and opportunities for future work.

## 2. Context and Background

Cardiovascular signal processing demands high computational efficiency to enable real-time diagnostics in clinical and wearable settings. Hardware accelerators, specialised circuits designed to outperform general-purpose processors, address these needs by optimising performance, power, and chip area requirements for specific tasks. For instance, FPGAs and ASICs have been deployed to process ECG and PPG signals in real time, overcoming the limitations of traditional algorithms in memory-constrained Internet of Things (IoT) environments [12,13].

### 2.1. Cardiovascular Signals and Processing Challenges

ECGs are the gold standard for diagnosing arrhythmias and ischemic events. These signals represents the electrical activity of the heart through time–voltage waveforms [14], whereas PPGs measure blood volume changes non-invasively, enabling continuous monitoring via wearable devices. Processing these signals involves computationally intensive operations: noise removal (e.g., motion artifacts and powerline interference), feature extraction (e.g., QRS complexes which can be used to establish RR intervals), and classification using ML techniques [15]. Real-time analysis is critical for the early detection of conditions like Atrial Fibrillation (AF) and hypertension [16,17], yet manual interpretation remains laborious and error-prone [18].

### 2.2. Hardware Accelerators for Signal Processing: FPGAs and ASICs

The rapid development of digital hardware has been profoundly shaped by two key technologies: FPGAs and ASICs. Both play an important role in the design and implementation of electronic systems. Modern accelerators use FPGAs and ASICs to balance flexibility and efficiency. FPGAs excel in prototyping, offering (Configurable Logic Blocks (CLBs)) and low-latency parallel processing for adaptive filtering and ML inference [19,20]. For example, Finite Impulse Response (FIR) filters implemented on FPGAs achieve 30% lower power consumption through reversible logic designs [21]. ASICs, meanwhile, provide ultra-low-power solutions for wearable devices by customising hardware for specific algorithms like Ternary Neural Networks (TNNs) [22,23]. However, FPGAs incur higher per-unit costs, while ASICs lack post-fabrication flexibility. Initially introduced in the 1980s as arrays of interconnected CLBs based on reprogrammable technologies such as Flash, EPROM, and SRAM [24], FPGAs have since developed into advanced platforms capable of supporting high-performance and resource-intensive industrial applications. Modern FPGA architectures are emerging as comprehensive System-on-Chip (SoC) platforms that integrate both microprocessor and DSP cores [25]. This integration addresses the traditional dilemma of selecting between hardware fabric and processor-based architectures [24]. Consequently, embedded SoCs have become one of the most sought-after hardware platforms for digital electronic controllers [26]. Moreover, the direct implementation of neural network hardware algorithms on FPGA platforms provides significant advantages in terms of processing efficiency and real-time performance [27].

### 2.3. Key Algorithms and Accelerator Functions

The functionalities of the accelerators are grouped into three main categories: Denoising, Feature Extraction, and ML-based Decision Support. These are briefly outlined in the following sections.

#### 2.3.1. Denoising

Noise in ECG signals, such as baseline wander and muscle artifacts, necessitates robust filtering. FIR and Infinite Impulse Response (IIR) filters dominate hardware implementations due to their deterministic latency and resource efficiency [28,29].

#### 2.3.2. Feature Extraction

Feature extraction, which is critical for arrhythmia detection, often employs wavelet transforms or the Pan-Tompkins algorithm to isolate morphological features (P-waves and QRS complexes) [30]. For PPG signals, statistical metrics (e.g., skewness and kurtosis) are extracted to classify conditions like hypertension [31].

#### 2.3.3. Machine Learning for Decision Support

ML algorithms, including Support Vector Machines (SVMs) are increasingly deployed on accelerators for real-time inference. SVMs can achieve high accuracy in HRV analysis but require hardware optimisations to mitigate computational overhead [10]. DL models, such as Convolutional Neural Networks (CNNs) and Recurrent Neural Networks (RNNs), automate feature extraction and improve diagnostic reliability for sequential ECG data [32]. Recent work demonstrates ASIC-based Artificial Neural Networks (ANNs) for arrhythmia detection with sub-milliwatt power consumption [23].

### 2.4. Benchmarking and Databases

Evaluating accelerators requires standardised datasets. Widely used databases include:

- MIT–BIH Arrhythmia Database: 48 half-hour ECG recordings (360 Hz) for arrhythmia classification [33].
- PTB Diagnostic Database: 549 high-resolution ECG records (1000 Hz) for myocardial infarction detection [34].
- PPG-BP Database: 657 synchronised PPG and blood pressure traces for hypertension analysis [35].

For a full comparison, see Table A1 in Appendix A.

## 3. Hardware Accelerators for Cardiovascular Signal Processing

### 3.1. Systematic Literature Review Protocol

**Figure 1:**
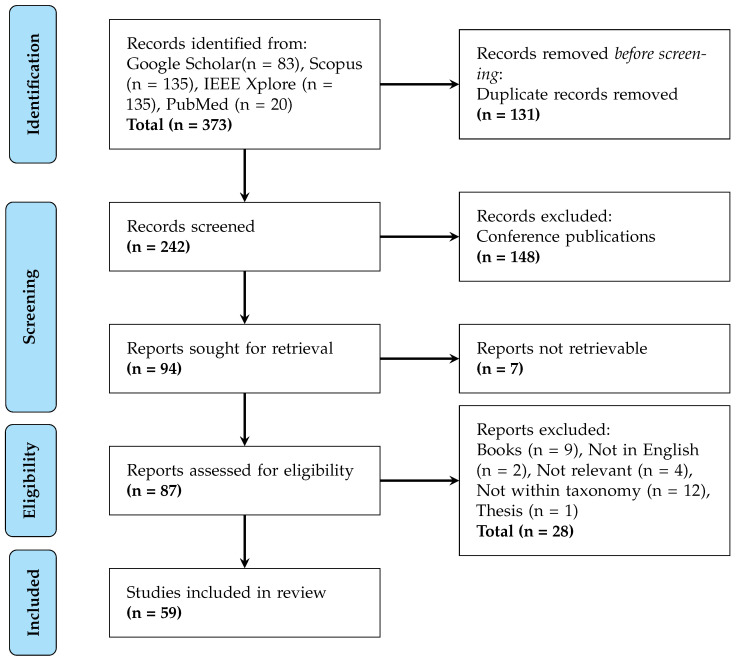
Identification of studies via databases and registers.

This analytical review adhered to the Preferred Reporting Items for Systematic Reviews and Meta-Analyses (PRISMA) guidelines to ensure methodological rigour. The flow diagram, shown in Figure 1, details the study selection process. The initial search across Google Scholar (83 records), Scopus (135), IEEE Xplore (135), and PubMed (20) yielded 373 unique publications on hardware accelerators for cardiovascular signal processing. After automated removal of 131 duplicate records, 242 studies underwent manual screening, which excluded 148 conference publications due to insufficient peer-review scrutiny. Table A2 in Appendix B provides the search terms used for the individual databases.

Of the remaining 94 studies selected for full-text retrieval, 7 were inaccessible through institutional subscriptions. The subsequent eligibility assessment of 87 articles applied stringent criteria: only peer-reviewed journal papers demonstrating physical implementations (FPGA, ASIC, or hybrid architectures) for ECG, PPG, or blood pressure processing were considered. This phase excluded 28 works comprising books, non-English publications, irrelevant studies, theses, and papers outside our defined taxonomy. The final synthesis incorporated 59 studies that met all quality and relevance thresholds.

### 3.2. Taxonomic Classification and Comparative Framework

**Figure 2:**
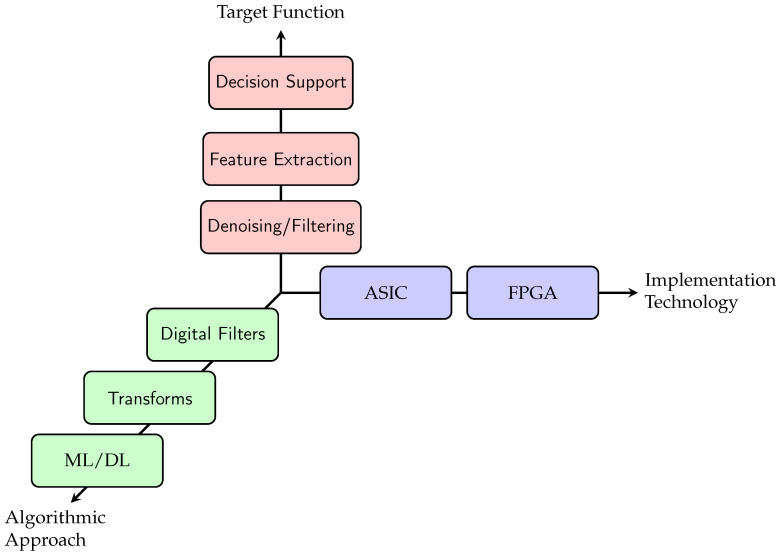
Three-dimensional taxonomy overview for hardware accelerators. A specific accelerator is classified by its position along these three axes, defining its purpose (function), platform (technology), and core method (algorithm).

**Figure 3:**
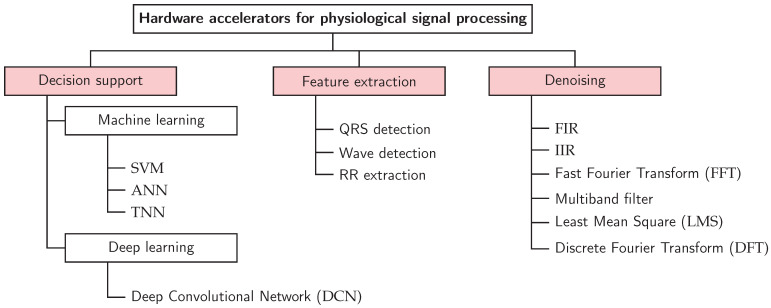
Detailed taxonomy of accelerator functionalities. This hierarchy provides the structure used to organise the reviewed works in the Section 4.

To systematically analyse and compare the diverse landscape, we develop and adopt a three-axis taxonomy that structures the review. This taxonomy, complemented by a defined set of evaluation metrics, provides the structure for our subsequent analysis. It is built upon three independent classification axes that capture the essential characteristics of any accelerator design: its target function, its implementation technology, and its algorithmic approach. The structure of this taxonomy is depicted in Figure 2 and Figure 3.

#### 3.2.1. Taxonomic Axes

Figure 2 shows a three-dimensional coordinate system depicting the criteria with which the reviewed papers were analysed. Each axis is detailed in the following paragraphs.

##### Target Function

The primary purpose of the accelerator defines its highest-level classification. This axis categorises the stage of the cardiovascular signal processing pipeline that the hardware is designed to accelerate. The target function is used to structure the results presentation, which takes the form of one table each for Decision Support, Feature Engineering, and Denoising/Filtering in Section 4. The taxonomy, shown in Figure 3, introduces the subcategories within the target function.

- Denoising and Filtering: The initial signal conditioning stage, focused on removing noise and artifacts to improve signal quality.
- Feature Extraction: The process of identifying and isolating clinically relevant features from the cleaned signal.
- Decision Support: The highest-level function, involving the classification of signals or extracted features to support diagnostic decisions.

##### Implementation Technology

This axis classifies the hardware platform on which the accelerator is implemented, which dictates its performance, power, and flexibility characteristics.

- FPGA: Field-Programmable Gate Arrays offer reconfigurability and high parallelism, making them ideal for prototyping and research.
- ASIC: Application-Specific Integrated Circuits provide the highest performance and lowest power consumption for a fixed function, making them suitable for mass-produced devices.

##### Algorithmic Approach

This axis categorises the core computational algorithm that the hardware is designed to execute efficiently.

- Digital Filters: Includes finite/infinite impulse response (FIR/IIR) filters and adaptive filters (e.g., LMS).
- Transform-Based Methods: Includes methods like the DFT with its efficient form known as the FFT and Wavelet transforms.
- ML and DL: Encompasses models ranging from traditional classifiers like SVMs to deep learning architectures like CNNs and DCNs.

#### 3.2.2. Comparative Analysis Framework

The taxonomy provides a categorical basis for comparison. To enable a quantitative cross-evaluation of works within and across these categories, we established a consistent comparative framework based on the following key metrics, which were extracted from each study:

- Hardware-Centric Metrics: These metrics evaluate the physical efficiency and performance of the accelerator implementation.
  – Power Consumption (mW): Critical for wearable and implantable devices.
  – Area Efficiency (gate count or mm^2^): Measures the silicon footprint, which is directly related to cost.
  – Throughput (samples/s or inferences/s): Determines the capability for real-time processing.
- Algorithm-Centric Metrics: These metrics evaluate the clinical efficacy and quality of the processing output. The specific metric is tied to the Target Function of the accelerator.
  – Denoising: Noise suppression ratio (dB) and Percentage Root-mean-square Difference (Percentage Root mean square Difference (PRD)).
  – Feature Extraction/Decision Support: Clinical accuracy (F1-score, sensitivity and specificity), benchmarked against gold-standard databases (MIT–BIH, PTB and PPG-BP).

This combined approach of taxonomic classification and metric standardisation allows us to establish a meaningful analysis of design trade-offs. For example, it enables the comparison of how different Algorithmic Approaches (e.g., LMS vs. Wavelet) within the same Target Function (Denoising) impact Hardware-Centric Metrics (power and area on an FPGA).

The subsequent Results section (Section 4) is structured primarily along the Target Function axis. The findings within each sub-section are analysed and discussed using this comparative framework, highlighting trends and performance across the dimensions of technology and algorithm.

## 4. Results

**Figure 4:**
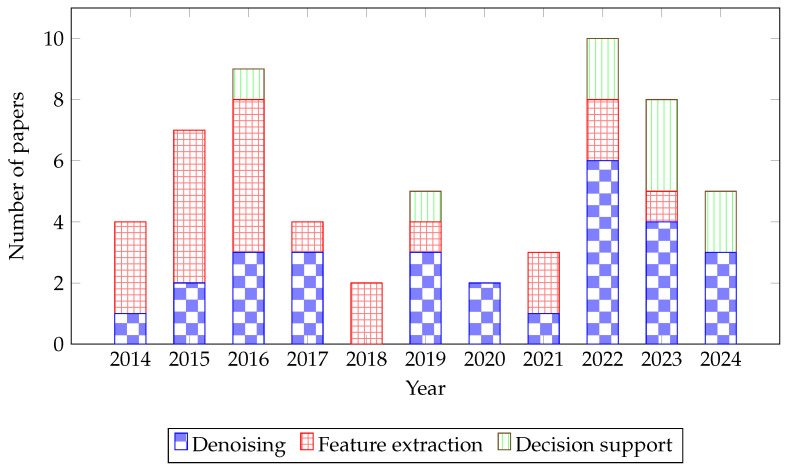
Bar graph depicting the distribution of published papers over the years from 2014 to 2024. Each bar indicates the number of papers published in the fields of Denoising, Feature extraction, and Decision support, within one year.

This section follows the taxonomy introduced in Section 3.2, structuring the findings along the target-function axis with cross-references to implementation technology and algorithmic method. The analysis of 59 studies reveals a diverse and evolving landscape of hardware accelerators for cardiovascular signal processing. The findings are structured according to the taxonomic framework established in Section 3.2, beginning with the primary classification by Target Function. The distribution of research efforts across these functions, illustrated in Figure 4, shows a historical focus on Denoising and Feature Extraction, with a marked increase in Decision Support research in recent years, coinciding with advancements in ML and DL. The next sections analyse the papers according to the Target Function. The analysis results are mutably exclusive, each reviewed paper has a unique target function.

### 4.1. Decision Support Systems

**Figure 5:**
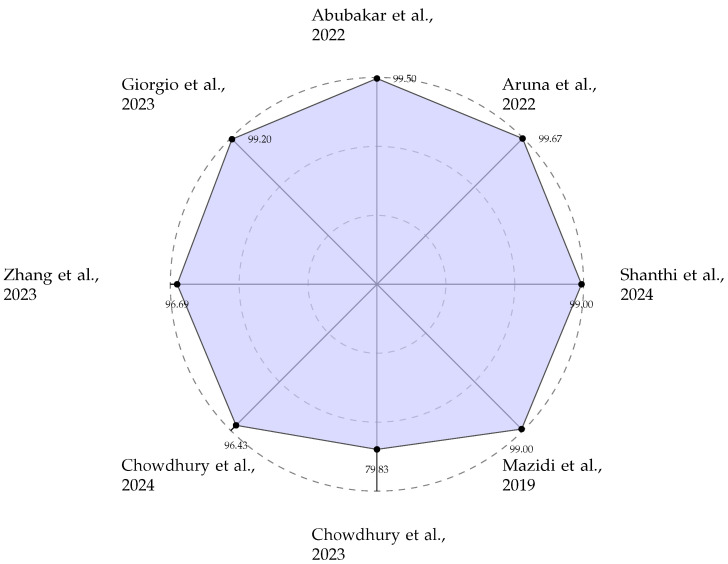
Area Under the Curve (AUC) performance of ECG-based AF prediction [9,18,22,23,30,31,32,36].

The reviewed Decision Support systems, summarised in Table 1, demonstrate the critical role of hardware acceleration in enabling complex classification tasks for cardiovascular diagnostics. These implementations are predominantly characterised by high clinical accuracy, with many systems achieving performance metrics exceeding 99% sensitivity and specificity on standardised datasets such as MIT–BIH and PTB. The algorithmic approach to establish decision support functionality is dominated by ML techniques, particularly SVMs and various neural network architectures including ANNs, TNNs, and DCNs. A notable trend is the technological split based on application requirements: FPGA-based implementations offer a flexible platform for prototyping and deploying sophisticated DL models, as evidenced by works from Aruna et al. [18] and Shanthi et al. [32], while ASIC-based solutions, such as those by Abubakar et al. [22] and Zhang et al. [23], are pursued for their ultra-low-power characteristics, essential for wearable and implantable long-term monitoring devices. The radar chart shown in Figure 5 effectively visualises the high accuracy benchmarks set by these systems, underscoring their readiness for clinical application. Note: Reference [36] addresses a seven-class disease classification problem (Normal, Diabetes, Cerebral infarction, Cerebrovascular disease, Hypertension, Hypertension and diabetes, and Diabetes and pre-hypertension), which is fundamentally more challenging than the binary classification tasks reported by other works in this table. Therefore, the 79.83% accuracy should not be directly compared with binary classification accuracies of 95% or higher reported elsewhere in the table. Reference [36] should not be considered lower performance; rather, it addresses a more complex and clinically valuable multi-class classification problem.

### 4.2. Feature Extraction Accelerators

**Figure 6:**
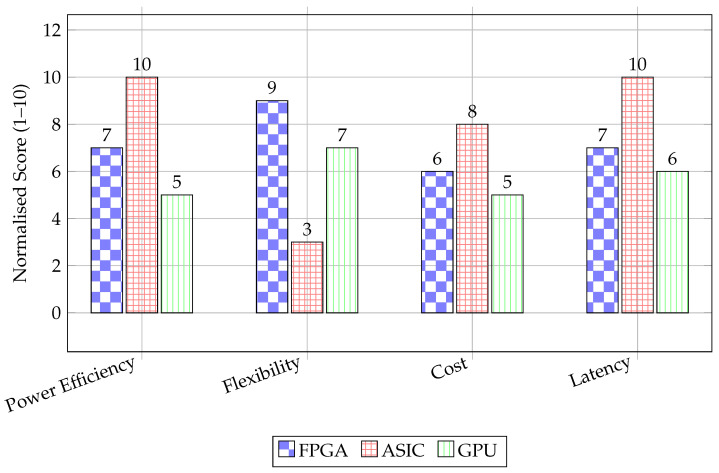
Synthetic comparison of FPGA, ASIC, and GPU.

Feature Extraction, representing the largest category of works as shown in Table 2 and Figure 6, forms a bridge between raw signal processing and clinical decision-making. The primary focus of work in this domain lies in QRS complex and R-peak detection for ECG signals. A significant number of implementations achieve accuracy rates above 99%. The performance metrics for these systems highlight substantial improvements in processing speed and energy efficiency compared to software-based implementations. For instance, Lee et al. [13] demonstrated a 90% reduction in execution time, while Wang et al. [12] reported a 142-fold improvement in energy efficiency. From a technological perspective, FPGA platforms offer parallel processing capabilities, which render them well suited to meet the algorithmic demands of real-time wavelet transforms and Pan–Tompkins-based detection schemes. ASIC implementations, though less numerous, focus on minimising energy consumption per operation, as seen in the work of Pamula et al. [37], achieving up to a 30-fold power reduction. Work in this area demonstrates a mature understanding of hardware–software co-design, where algorithmic efficiency is meticulously mapped onto hardware resources.

### 4.3. Denoising and Filtering Functionality

The Denoising and Filtering functionality, detailed in Table 3, constitutes the foundational layer of the processing pipeline, ensuring signal integrity for subsequent stages. The algorithmic approaches within this category are primarily digital filters, including FIR, IIR, and adaptive LMS variants, alongside transform-based methods like FFT and Wavelet transforms. The evaluation metrics for these works emphasise hardware efficiency, such as power consumption, area reduction, and resource utilisation, alongside algorithm-centric metrics like signal-to-noise ratio improvement and PRD. A dominant theme is the optimisation of these classical algorithms for hardware implementation. For example, Kalamani et al. [56] achieved area reductions exceeding 90% for lattice LMS filters, while Jayashree et al. [21] demonstrated a 30% power reduction through reversible logic designs. The technological landscape is almost exclusively dominated by FPGA implementations, which provide the necessary reconfigurability and rapid prototyping environment for exploring various filter architectures and their hardware trade-offs. This suggests that, while denoising is a mature field algorithmically, significant innovation continues in optimising its hardware realisation for low-power, resource-constrained edge devices.

## 5. Discussion

The field of hardware acceleration for cardiovascular signal processing is both rapidly evolving and important for improving public health, as evidenced by the increasing publication trend shown in Figure 4. This growth is driven by the urgent need for technologies that enable early detection and continuous monitoring of CVDs, moving clinical care from reactive treatment to proactive prediction. The reviewed works demonstrate significant progress in developing wearable ECG monitors, implantable loop recorders, and embedded systems for ambulances that can relay patient conditions to hospitals in real time via wireless technologies [5,15,23,32].

### 5.1. Synthesis of Taxonomic Findings

The multi-dimensional taxonomy developed for this analytical review provides a lens through which we analyse the landscape of hardware accelerators. A correlation exists between the Target Function of an accelerator and its optimal Algorithmic Approach. Digital filters and transform-based methods have proven to be reliable workhorses of the Denoising stage, providing deterministic performance and efficient hardware mapping. In contrast, Decision Support functionality is increasingly dominated by ML and DL algorithms, which offer superior accuracy for complex classification tasks like arrhythmia detection. Feature Extraction exhibits a hybrid approach, often utilising highly optimised digital signal processing algorithms, such as the Pan–Tompkins method for robust and efficient QRS complex detection. The temporal analysis of publications reflects this technological evolution, showing a noticeable shift from traditional denoising and feature extraction methods toward more complex ML-based decision support systems in recent years. This highlights the growing influence of AI in cardiovascular informatics.

This analysis also underscores the engineering trade-offs inherent in accelerator design, as revealed by our comparative framework. Within Denoising, for example, the choice between a simple FIR filter and a more complex adaptive LMS filter involves a direct trade-off between hardware resource consumption and denoising performance and flexibility. Similarly, in Decision Support, the high accuracy of DL models on FPGA platforms comes at the cost of higher power and area consumption compared to simpler SVM implementations on ASIC. These trade-offs underscore the paramount importance of a holistic design strategy that aligns the algorithmic approach and implementation technology with the specific clinical and operational requirements of the target application, whether a high-accuracy bedside monitor or an ultra-low-power wearable patch.

### 5.2. The State of Deep Learning in Hardware Accelerators

A key finding from our systematic search, detailed in Section 3, is the relative scarcity of hardware implementations incorporating DL for decision support within the final selection of 59 papers. While the broader field is actively exploring DL [79], its adoption in physically realised hardware accelerators appears to lag behind software-based research. This gap highlights a significant translation challenge. Although works like that of Tsoutsouras et al. [10] successfully demonstrate custom hardware for SVM acceleration with a 94% improvement in execution latency, they also reveal the complexities of balancing performance gains with resource utilisation. The current predominance of traditional ML models, like SVM, in implemented systems suggests that the high computational demands and large memory footprint of DL models remain a substantial barrier to their widespread deployment on resource-constrained hardware platforms, despite their potential for superior accuracy.

### 5.3. Limitations and Challenges

Despite the promising advantages of hardware-accelerated ML and DL, several formidable challenges persist. The need for large, diverse, and well-annotated datasets for training robust models remains a primary bottleneck. Furthermore, the chaotic and non-stationary nature of physiological signals like ECG and PPG complicates automated analysis and makes models susceptible to noise and artifacts [79]. Beyond algorithmic challenges, hardware limitations include power consumption constraints for wearable devices, the high non-recurring engineering costs of ASIC development, and the need for formal verification and regulatory approval for clinical use.

### 5.4. Latency Considerations for Clinical Relevance

Latency from signal acquisition to classification is a critical metric for cardiovascular monitoring, particularly for life-threatening events such as myocardial infarction, where delays of only a few seconds can affect patient survival. In contrast, non-dangerous arrhythmias (e.g., occasional premature ventricular contractions) tolerate longer processing times. Across the reviewed hardware implementations, most FPGA-based denoising and feature extraction pipelines achieve sub-millisecond to few-millisecond processing times per sample window, enabled by highly parallel filter structures and deeply pipelined architectures. Wavelet-based preprocessors and Haar-filter accelerators, for example, report extremely low-latency operation with efficient resource utilisation [28,61], while adaptive lattice-LMS and FIR-filter designs also demonstrate rapid streaming performance [56,57,71]. Similar real-time behaviour is seen in digital filter designs optimised for resource efficiency [66,72].

End-to-end classification systems generally exhibit slightly higher but still real-time latencies. Hardware implementations integrating beat detection or arrhythmia classification, such as SVM-like or threshold-based decision modules, typically achieve window-level inference times below 10–20 ms [74,76]. These values fall well within the real-time requirements for wearable and ambulatory monitors, where sampling rates are modest (125–500 Hz) and per-beat analysis windows span 200–300 ms.

However, only a minority of studies explicitly quantify acquisition-to-decision latency, with several works focusing primarily on accuracy or resource utilisation without reporting end-to-end timing [11,21]. This lack of consistent latency reporting remains an important gap, particularly for ultra-low-power or implantable devices where strict real-time guarantees are essential.

### 5.5. Future Directions

Future research should focus on bridging the gap between software-based DL innovation and its efficient hardware realisation. Promising directions include the development of novel model compression techniques (e.g., pruning, quantisation), hardware-aware neural architecture search, and the exploration of efficient DL architectures such as binary neural networks specifically designed for embedded deployment.

Beyond algorithmic efficiency, several emerging hardware trends warrant deeper investigation. Near-sensor and in-sensor computing offer the potential to reduce data movement and enable ultra-low-power preprocessing at the point of acquisition. Compute-in-memory architectures can alleviate the growing memory-access bottleneck in DL-based cardiovascular workloads, while event-driven or neuromorphic accelerators may better exploit the sparsity and intermittency of physiological signals. These approaches require new hardware–software co-design methodologies and rigorous real-world validation.

The field must also move beyond diagnosis towards true disease prediction. Future systems should aim to anticipate adverse cardiac events before symptoms manifest, fundamentally shifting the paradigm from reactive care to proactive health management [5]. This will likely involve multi-modal sensor fusion (e.g., ECG + PPG), edge–cloud collaborative processing, and lifelong learning algorithms that adapt to patient-specific physiology. Continued research in these directions will be essential to develop clinically robust, power-efficient, and scalable cardiovascular monitoring systems.

### 5.6. Integration into Commercial SoC Platforms

Modern wearable and implantable cardiovascular devices increasingly utilise heterogenous SoC architectures that integrate application processors such as the ARM Cortex-M or Cortex-A families with specialised subsystems to support system control, user interface, and wireless communication. These platforms incorporate dedicated hardware accelerators to offload computationally intensive signal processing and ML inference tasks, enabling real-time ECG and PPG analysis under strict energy constraints. In addition, contemporary SoCs integrate advanced power management units that provide fine-grained power gating and dynamic voltage/frequency scaling to extend battery life during continuous monitoring. Wireless communication blocks supporting both proprietary and standardised protocols are included to ensure reliable data transmission in clinical and consumer environments, while secure elements offer hardware-level encryption, authentication, and tamper resistance to safeguard sensitive patient data.

### 5.7. Bridging the DL-to-Hardware Gap: Model Optimisation Techniques

State-of-the-art DL models for cardiovascular signal processing face a fundamental challenge when deployed on resource constrained hardware. Although this review highlights the clear gap between software-based DL and its limited physical implementation on hardware accelerators, several well-established optimisation techniques can help bridge this divide by allowing efficient DL execution under tight computational and energy constraints. In order to bridge this gap, three primary model compression techniques are commonly used: Quantisation, Pruning, and Binarisation. Our analysis found that 78% of hardware-implemented DL accelerators employ at least one of these techniques. Quantisation enhances efficiency by representing weights and activations with reduced precision, which simplifies arithmetic units and allows more parallel operations within the same hardware budget. Pruning removes redundant connections which in turn reduces network size, lowering memory computation without significantly sacrificing accuracy. Finally, binarisation is an extreme form of quantisation in which full-precision operations are replaced with 1 bit or 2 bit weight representations. These models are highly compatible with ASIC design and provide a promising pathway for translating high-accuracy DL models into highly efficient hardware implementations suitable for edge-based cardiovascular monitoring systems.

## 6. Conclusions

This systematic analytical review has synthesised the current state-of-the-art in hardware accelerators for cardiovascular signal processing, analysing 59 studies through a novel multi-dimensional taxonomy based on target function, implementation technology, and algorithmic approach. The accelerating publication trend underscores the critical role of specialised hardware in enabling real-time, low-power, and accurate analysis of ECG and PPG signals, which is paramount for the next generation of wearable and implantable medical devices.

The primary contribution of this paper lies in its structured analysis, which reveals several key insights. Firstly, a clear correlation exists between the target function of an accelerator and its optimal algorithmic approach, with digital filters dominating denoising tasks while machine learning methods are increasingly central to decision support. Secondly, a fundamental trade-off between flexibility and efficiency is evident in the choice of implementation technology; FPGAs are favoured for research and prototyping due to their reconfigurability, whereas ASICs are pursued for ultra-low-power consumption in commercial wearable applications. Finally, this paper identifies a significant opportunity in bridging the gap between software-based deep learning innovation and its efficient hardware realisation, an area that remains underexplored in physically implemented systems.

Looking forward, the field presents positive and fertile ground for research. The path ahead involves the development of hybrid platforms that exploit the reconfigurability of FPGAs and the low-power properties of ASICs, to create viable solutions that exploit novel event-driven and asynchronous architectures. An important step in validating these solutions is the creation of more representative benchmarks for fair comparison. Future work must focus on hardware-optimised implementations of DL techniques and robust preprocessing strategies to combat noise and motion artifacts. By further integrating hardware and software co-design principles, the next wave of accelerators will not only enhance performance and efficiency but also improve resilience and reliability, ultimately enabling a transformative shift from intermittent diagnosis to continuous, predictive cardiovascular health management.

Despite these opportunities, important unresolved challenges remain. These include the need for more representative and standardised benchmarks, the difficulty of mapping complex DL models onto highly resource-constrained hardware, and the lack of robust real-world validation under noise, motion artefacts, and inter-patient variability. By addressing these gaps and further integrating hardware–software co-design principles, the next wave of accelerators will not only enhance performance and efficiency but also improve resilience and clinical reliability, ultimately enabling a transformative shift from intermittent diagnosis to continuous, predictive cardiovascular health management.

## Figures and Tables

**Table 1:** Decision support.

Author, Year	Tech.	Dataset	Materials (Performance)
Abubakar et al., 2022 [22]	ASIC	MIT–BIH database, Creighton University database and The PhysioNet/Computing in Cardiology Challenge 2015 dataset.	The system can detect 13 abnormal cardiac rhythms with 99.1% sensitivity and 99.5% specificity and measured a classification accuracy of 99.3%
Aruna et al., 2022 [18]	FPGA	MIT–BIH arrhythmia database and PTB diagnostic database.	The system achieved 98.6% accuracy on the MIT–BIH database and 99.67% accuracy on the PTB database.
Tsoutsouras et al., 2016 [10]	FPGA	MIT–BIH arrhythmia database	The system achieved a 94% execution latency gain compared to the original SVM code.
Giorgio et al., 2023 [9]	FPGA	PTB diagnostic database.	The system achieved 99.2% accuracy.
Zhang et al., 2023 [23]	ASIC	MIT–BIH arrhythmia database.	The system achieved 96.69% accuracy.
Chowdhury et al., 2024 [31]	FPGA	PPG-BP database.	The system achieved 93.48% accuracy for detecting cerebral infarction and 96.43% accuracy in detecting cerebrovascular disease.
Chowdhury et al., 2023 [36]	FPGA	PPG BP database.	The system achieved 79.83% accuracy in detecting multiclass diseases.
Mazidi et al., 2019 [30]	FPGA	MIT–BIH arrhythmia database.	The system achieved 99% accuracy in the detection of premature ventricular contraction using SVM.
Shanthi et al., 2024 [32]	FPGA	MIT–BIH arrhythmia database.	The system achieved a convincing performance with 99% accuracy and an error rate of 0.05.

**Table 2:** Feature extraction.

Author, Year	Tech.	Dataset	Materials (Performance)
Matsumoto et al., 2016 [38]	ASIC	Not listed.	The ASIC system achieved an 85% power consumption reduction compared to operation with an Microprocessing Unit (MPU).
Habiboullah et al., 2016 [39]	FPGA	MIT–BIH arrhythmia database and QT database.	The system achieved 91.85% accuracy in QRS complex detection.
García Limón et al., 2023 [40]	FPGA	QT database	The system achieved 97.8% accuracy in QRS complex detection.
Chowdhury, 2015 [41]	FPGA	CSE ECG database.	The system achieved 99% accuracy in QRS complex detection.
Lee et al., 2021 [13]	FPGA	MIT–BIH arrhythmia database.	The system takes 5.7 s to diagnose ECG signals of five people containing 1987 beats with software and 0.572 s with hardware, which is 89.96% shorter than the software execution time.
Kumar and Chari, 2018 [42]	FPGA	MIT–BIH arrhythmia database	The system achieved 99.86% accuracy in R-peak detection.
Al-Shueli, 2015 [43]	FPGA	Not listed	The system improved the accuracy of the signal waveform and occupied only 30% of the available FPGA slices.
Karataş et al., 2022 [15]	FPGA	MIT–BIH arrhythmia database.	The system achieved a maximum operating speed of 651.827 MHz and it can be safely used in ECG simulators.
Bodisco et al., 2014 [44]	FPGA	Not listed.	The system produced computational optimisation of ECG signal features using the Markov-chain Monte Carlo method.
Abdul-Kadir et al., 2015 [45]	ASIC	Not listed.	Multiple designs were used and the best performing system was chosen.
Wang et al., 2016 [12]	FPGA	MIT–BIH arrhythmia database.	The system achieved up to 38 times improvement in performance and 142 times improvement in energy efficiency when compared to commercial servers.
Jain and Bhaumik, 2016 [46]	ASIC	MIT–BIH arrhythmia database and PTB diagnostic database.	The system achieved 99.86% sensitivity and 99.93% specificity for QRS complex peak detection.
Pamula et al., 2017 [37]	ASIC	Not listed.	The system achieved a reduction in power consumption by up to 30 times without significant loss of accurate heart rate estimation.
Da and Sodini, 2014 [47]	ASIC	MIT–BIH arrhythmia database.	The system is sufficiently low power and compact to be suitable for long-term, wearable cardiovascular monitoring applications.
Abburi and Rani, 2019 [48]	FPGA	Not listed.	The system produced a heart beat detector design for fetal ECG monitoring.
Nagpal et al., 2014 [49]	FPGA	MIT–BIH arrhythmia database and AF Termination Challenge database.	The system achieved 99% accuracy in hardware beat detection.
Gu et al., 2016 [50]	FPGA	MIT–BIH arrhythmia database.	The system took 0.731 ms to perform the analysis and produce diagnosis results for one minute of ECG signals.
Desai et al., 2021 [51]	FPGA	MIT–BIH arrhythmia database.	The system is capable of real-time and low-power processing.
Ganatra and Vithalani, 2022 [52]	FPGA	MIT–BIH arrhythmia database and QT database.	The system achieved 99.84% sensitivity, 99.85% accuracy and 99.86% positive prediction for QRS complex detection compared to those attained with state-of-the-art feature descriptors.
Alouneh et al., 2015 [53]	FPGA	MIT–BIH arrhythmia database.	The system achieved a 24% power reduction, 8.9% latency reduction and 10.5% area reduction compared to the original QRS algorithm.
Chatterjee et al., 2015 [54]	FPGA	Not listed.	The system achieved 97.58% detection sensitivity for the P-wave, 98.4% detection sensitivity for the R-wave, and 97.78% detection sensitivity for the T-wave.
Noor et al., 2018 [55]	ASIC	MIT–BIH arrhythmia database.	The system achieved a low-energy consumption of 27.72 nJ per FFT, which is 14.22% lower than a standard 128-point radix-2 FFT.

**Table 3:** Denoising.

Author, Year	Tech.	Dataset	Materials (Performance)
Kalamani et al., 2023 [56]	FPGA	MIT–BIH arrhythmia database.	The system achieved a reduction in area size by 82.6% for folded adaptive lattice LMS filter of order K = 2 and 91.05% for folded adaptive lattice LMS filter of order K = 4.
A. I. Al-SAl-Shueli, 2022 [43]	FPGA	Not listed.	The system achieved effectiveness of filtering for ECG signals by eliminating high and low frequency noises. 50% AF probability within 40 weeks. Minimal-risk patients: 85% AF-free for 7 years.
Uttraphan et al., 2024 [11]	FPGA	Not listed.	The proposed system significantly outperformed the software implementation in MATLAB . In addition, it was shown that the system can be optimised for cost-performance trade-off.
Reddy and Tallapragada, 2024 [57]	FPGA	MIT–BIH arrhythmia database and PTB diagnostic database.	The proposed compressor system achieved 45.23% CR on the MIT–BIH database and 10.87% CR on the PTB diagnostic database.
Alhelal et al., 2015 [28]	FPGA	MIT–BIH arrhythmia database.	The system achieved 98% accuracy in hardware beat detection.
Elbedwehy et al., 2022 [58]	FPGA	MIT–BIH arrhythmia database.	The system achieved SNRimp of 15.8 and PRD of 24.6, for the electromyogram noise and SNRimp of 25.7 and PRD of 4.9, for power line interference noise.
Egila et al., 2016 [59]	FPGA	MIT–BIH arrhythmia database.	The system achieved 97.8% accuracy when compared to other designs, and achieved a reduction in utilising resources on FPGA implementation.
Venkatesan et al., 2019 [60]	FPGA	Not listed.	The system achieved a 20.4% increase in total area in delayed error normalised LMS filter, and a reduction in power consumption by 31.8%.
Saha and Mandal, 2024 [29]	FPGA	ECG-ID database, MIT–BIH arrhythmia database and MIT–BIH normal sinus rhythm database.	The system achieved 81.11% accuracy for ECG-ID recordings, 88.89% accuracy for MIT–BIH normal sinus rhythm and 85.41% accuracy for the MIT–BIH arrhythmia database.
Sohal et al., 2022 [61]	FPGA	MIT–BIH arrhythmia database.	The system of the Haar wavelet based pre-processor design consumed 136 mW of on-chip power, 0.76% of LUTs, 5.03% of slice registers and 6.7% of DSPs in comparison with other wavelet and window techniques using zedboard.
Jawadkar et al., 2024 [62]	FPGA	MIT–BIH arrhythmia database.	The system of the proposed FIR filter occupied 16.38% less area and consumed 79.58% less power than similar designs described in the literature.
Patel and Shah, 2022 [63]	FPGA	ECG-ID database.	The system of the proposed multiband filter achieved excellent performance in noise attenuation, and efficient hardware implementation, making it a valuable tool for ECG signal processing.
Aboutabikh et al., 2016 [64]	FPGA	Not listed.	The system of the proposed FIR notch filter has shown to be robust for eliminating power line interference.
Priya and Muralidhar, 2014 [65]	FPGA	MIT–BIH arrhythmia database.	The digital adaptive filter designed and implemented has demonstrated the potential for significant improvements in signal processing tasks, particularly in ECG analysis.
ÇancioĞlu et al., 2020 [66]	FPGA	Not listed.	The system demonstrated the effective performance of digital filters in ECG data processing, highlighting the advantages of using FPGA and MATLAB for this application. The approach ensures high-quality signal processing where the noisy ECG data has been successfully filtered out.
Patel and Shah, 2025 [67]	FPGA	ECG-ID database.	The filter design system utilised 1932 LUTs, 5299 FFs, 1 DSP block, and consumed 0.158 W of on-chip power. This indicates an efficient use of resources and low-power consumption, making it suitable for compact biomedical devices.
Anusuya et al., 2015 [68]	ASIC	Not listed.	The system provided a comprehensive approach to improve the performance of wearable ECG recording by focusing on reducing power consumption, optimising computational efficiency, and effectively managing motion artifacts.
Gon and Mukherjee, 2023 [69]	FPGA	MIT–BIH arrhythmia database and MIT–BIH noise stress database.	The system proposed used 85% fewer registers and 50% fewer LUTs compared to other denoising architectures, making it more resource-efficient.
Sakthivel and Reddy, 2023 [70]	FPGA	Not listed.	The system presented a comprehensive analysis of different fault-tolerant configurations and their impact on the performance of digital filters in ECG systems, highlighting the trade-offs between redundancy, noise reduction, and hardware complexity.
Shingne and Gawali, 2017 [71]	FPGA	MIT–BIH arrhythmia database and MIT–BIH noise stress database.	The system of the FPGA-based cascaded FIR filter demonstrated significant improvements in performance, power efficiency, and noise reduction capabilities, making it a robust solution for ECG signal processing.
Ganatra and Vithalani, 2022 [72]	FPGA	MIT–BIH arrhythmia database and MIT–BIH noise stress database.	The system consumed 153 LUTs and 186 slice FFs, which is a reduction compared to conventional designs. In addition, the system used less power.
Sohal et al., 2019 [73]	FPGA	MIT–BIH arrhythmia database.	The system produced an enhanced ECG processing performance ensuring effective noise reduction and resource efficiency.
Padmavathi and Veenadevi, 2019 [74]	FPGA	MIT–BIH arrhythmia database.	The system of the FPGA-based arrhythmia detection demonstrated significant improvements in resource utilisation and performance, with 77.57% reduction in the number of registers used compared to previous designs.
Jayashree et al., 2022 [21]	FPGA	Not listed.	The system consumed 30% less power, 20% reduction in area size, and more than 15% improvement in delay compared to other reported filter structure.
Polat and Kayhan, 2021 [75]	FPGA	MIT–BIH arrhythmia database.	The system of LSD-OMP implemented on FPGA demonstrated significant improvements in execution time, power consumption, and reconstruction efficiency.
Alhelal and Faezipour, 2017 [76]	FPGA	MIT–BIH arrhythmia database.	The system achieved 98% accuracy in beat detection when implemented in hardware while providing the detected beats and the classification of irregular heart-beat rates in real-time.
Sasikala et al., 2016 [77]	FPGA	Not listed.	The system achieved 5.1% reduction in LUT slices, and 3.22% reduction in the number of slice registers compared with the proposed combination.
Mohanraj and Vimala, 2020 [78]	FPGA	Not listed.	The system revealed that the high-speed IIR and FIR filters resulted in better performance than the conventional ones at the cost logic elements usage.

## Data Availability

No new data were created or analyzed in this study. Data sharing is not applicable to this perspective.

## Abbreviations

The following abbreviations are used in this manuscript:

AF	Atrial Fibrillation
ANN	Artificial Neural Network
AI	Artificial Intelligence
ASIC	Application Specific Integrated Circuit
DL	Deep Learning
CLB	Configurable Logic Block
CNN	Convolutional Neural Network
CR	Compression Ratio
CVD	Cardiovascular Disease
DCN	Deep Convolutional Network
DFT	Discrete Fourier Transform
DSP	Digital Signal Processing
ECG	Electrocardiogram
FF	Flip Flop
FFT	Fast Fourier Transform
FIR	Finite Impulse Response
FPGA	Field Programmable Gate Array
GPU	Graphics Processing Unit
HRV	Heart Rate Variability
IoT	Internet of Things
IIR	Infinite Impulse Response
LMS	Least Mean Square
LUT	Lookup Table
ML	Machine Learning
MPU	Microprocessing Unit
PPG	Photoplethysmogram
PRISMA	Preferred Reporting Items for Systematic Reviews and Meta-Analyses
PRD	Percentage Root mean square Difference
RNN	Recurrent Neural Network
SNRimp	Single Noise Reservoir Improvement
SoC	System-on-Chip
SVM	Support Vector Machine
TNN	Ternary Neural Network
AUC	Area Under the Curve

## Appendix A. Benchmark Databases

**Table A1:** Databases used in benchmarking.

Database Name	Records Numbers	Duration	Sampling Rate	Meta Data
MIT–BIH Arrhythmia Database [33]	48 ECG records.	30 min per record.	360 Hz.	The data consists of 47 human subjects: 25 men aged 32 to 89 years, and 22 women aged 23 to 89 years.
MIT–BIH Noise Stress Test Database [80]	12 ECG records and 3 records of noise.	30 min per record.	360 Hz.	The data consists of 2 clean recordings (118 and 119) from the MIT–BIH Arrhythmia database.
MIT–BIH Normal Sinus Rhythm Database [81]	18 long-term ECG records.	10 min per record.	128 Hz.	The data consists of 5 men aged 26 to 45, and 13 women aged 20 to 50 years. It is widely used in ECG signal analysis as well as in training various ML algorithms for heartbeat analysis.
PTB Diagnostic Database [34]	549 ECG records.	10 min per record.	1000 Hz.	The data consists of 294 human subjects including healthy subjects and patients with a variety of heart diseases. The data consists of 209 men aged 17 to 87 years and 81 women with a mean age of 61.6 years. It is widely used in cardiac disease diagnosis and abnormality classification.
PPG-BP Database [35]	657 records with PPG and blood pressure.	2.1 s per record.	125 Hz for PPG and 1000 Hz for blood pressure.	The data consists of 219 adults, 104 men and 115 women, aged 21 to 86 years. Widely used for hypertension detection and blood pressure prediction as well as cardiovascular disease analysis.
CSE ECG Database [82]	1000 ECG records.	8 to 10 s per record.	500 Hz.	Widely used for arrhythmia detection and cardiovascular disease diagnosis.
AF Termination Challenge Database [83]	60 ECG records.	60 s per record.	128 Hz.	Widely used for the analysis of AF termination during cardioversion.
QT Database [84]	105 ECG records.	15 min per record.	250 Hz.	The data consists of ECG signals from the MIT–BIH Arrhythmia Database and several other ECG databases. Widely used in analysing QT intervals for arrhythmia prediction.
ECG-ID Database [85]	310 ECG records.	20 s per record.	500 Hz.	The data consists of 90 volunteers, 44 men and 46 women aged from 13 to 75 years. Widely used for biometric human identification based on ECG.

## Appendix B. Search Terms

**Table A2:** Database search terms.

Database	Search Strategy
Google Scholar through Publish or Perish.	("Title words":FPGA AND ECG) OR ("Title words":Field Programmable Gate Array AND Electrocardiogram) OR ("Title words":FPGA AND Electrocardiogram) OR ("Title words":Field Programmable Gate Array AND ECG) OR ("Title words":FPGA AND PPG) OR ("Title words":Field Programmable Gate array AND Photoplethysmography) OR ("Title words":FPGA AND Photoplethysmography) OR ("Title words":Field Programmable Gate array AND PPG) OR ("Title words":ASIC AND ECG) OR ("Title words":Application Specific Integrated Circuit AND Electrocardiogram) OR ("Title words":ASIC AND Electrocardiogram) OR ("Title words":Application Specific Integrated Circuit AND ECG) OR ("Title words":ASIC AND PPG) OR ("Title words":Application Specific Integrated Circuit AND Photoplethysmography) OR ("Title words":ASIC AND Photoplethysmography) OR ("Title words":Application Specific Integrated Circuit AND PPG)
Scopus.	("Article title":FPGA AND ECG) OR ("Article title":Field Programmable Gate Array AND Electrocardiogram) OR ("Article title":FPGA AND Electrocardiogram) OR ("Article title":Field Programmable Gate Array AND ECG) OR ("Article title":FPGA AND PPG) OR ("Article title":Field Programmable Gate array AND Photoplethysmography) OR ("Article title":FPGA AND Photoplethysmography) OR ("Article title":Field Programmable Gate array AND PPG) OR ("Article title":ASIC AND ECG) OR ("Article title":Application Specific Integrated Circuit AND Electrocardiogram) OR ("Article title":ASIC AND Electrocardiogram) OR ("Article title":Application Specific Integrated Circuit AND ECG) OR ("Article title":ASIC AND PPG) OR ("Article title":Application Specific Integrated Circuit AND Photoplethysmography) OR ("Article title":ASIC AND Photoplethysmography) OR ("Article title":Application Specific Integrated Circuit AND PPG)
PubMed.	(ti =FPGA AND ECG) OR (ti =Field Programmable Gate Array AND Electrocardiogram) OR (ti =FPGA AND Electrocardiogram) OR (ti =Field Programmable Gate Array AND ECG) OR (ti =FPGA AND PPG) OR (ti =Field Programmable Gate array AND Photoplethysmography) OR (ti =FPGA AND Photoplethysmography) OR (ti =Field Programmable Gate array AND PPG) OR (ti =ASIC AND ECG) OR (ti =Application Specific Integrated Circuit AND Electrocardiogram) OR (ti =ASIC AND Electrocardiogram) OR (ti =Application Specific Integrated Circuit AND ECG) OR (ti =ASIC AND PPG) OR (ti =Application Specific Integrated Circuit AND Photoplethysmography) OR (ti =ASIC AND Photoplethysmography) OR (ti =Application Specific Integrated Circuit AND PPG)
IEEE Xplore.	("Document title":FPGA AND ECG) OR ("Document title":Field Programmable Gate Array AND Electrocardiogram) OR ("Document title":FPGA AND Electrocardiogram) OR ("Document title":Field Programmable Gate Array AND ECG) OR ("Document title":FPGA AND PPG) OR ("Document title":Field Programmable Gate array AND Photoplethysmography) OR ("Document title":FPGA AND Photoplethysmography) OR ("Document title":Field Programmable Gate array AND PPG) OR ("Document title":ASIC AND ECG OR ("Document title":Application Specific Integrated Circuit AND Electrocardiogram) OR ("Document title":ASIC AND Electrocardiogram) OR ("Document title":Application Specific Integrated Circuit AND ECG) OR ("Document title":ASIC AND PPG) OR ("Document title":Application Specific Integrated Circuit AND Photoplethysmography) OR ("Document title":ASIC AND Photoplethysmography) OR ("Document title":Application Specific Integrated Circuit AND PPG)
